# A Comprehensive Study of a Micro-Channel Heat Sink Using Integrated Thin-Film Temperature Sensors

**DOI:** 10.3390/s18010299

**Published:** 2018-01-19

**Authors:** Tao Wang, Jiejun Wang, Jian He, Chuangui Wu, Wenbo Luo, Yao Shuai, Wanli Zhang, Xiancai Chen, Jian Zhang, Jia Lin

**Affiliations:** 1 School of Microelectronics and Solid-State Electronics, University of Electronic Science and Technology of China, North Jianshe Road, Chengdu 610054, China; wstudy92@163.com (J.W.); jackyhale@163.com (J.H.); cgwu@uestc.edu.cn (C.W.); luowb@uestc.edu.cn (W.L.); yshuai@uestc.edu.cn (Y.S.); wlzhang@uestc.edu.cn (W.Z.); 2 State Key Laboratory of Electronic Thin Film and Integrated Devices, University of Electronic Science and Technology of China, North Jianshe Road, Chengdu 610054, China; 3 The 29th Research Institute of China Electronics Technology Group Corporation, 496 West Yingkang Road, Chengdu 610036, China; chenxiancai_29@126.com (X.C.); mse_zhj@163.com (J.Z.); jialin_29@foxmail.com (J.L.)

**Keywords:** micro-channel heat sink, power IC, thin film temperature sensors, microfluidics, heat dissipation

## Abstract

A micro-channel heat sink is a promising cooling method for high power integrated circuits (IC). However, the understanding of such a micro-channel device is not sufficient, because the tools for studying it are very limited. The details inside the micro-channels are not readily available. In this letter, a micro-channel heat sink is comprehensively studied using the integrated temperature sensors. The highly sensitive thin film temperature sensors can accurately monitor the temperature change in the micro-channel in real time. The outstanding heat dissipation performance of the micro-channel heat sink is proven in terms of maximum temperature, cooling speed and heat resistance. The temperature profile along the micro-channel is extracted, and even small temperature perturbations can be detected. The heat source formed temperature peak shifts towards the flow direction with the increasing flow rate. However, the temperature non-uniformity is independent of flow rate, but solely dependent on the heating power. Specific designs for minimizing the temperature non-uniformity are necessary. In addition, the experimental results from the integrated temperature sensors match the simulation results well. This can be used to directly verify the modeling results, helping to build a convincing simulation model. The integrated sensor could be a powerful tool for studying the micro-channel based heat sink.

## 1. Introduction

With the development of microelectronics technology, transistors in an integrated circuit (IC) keep scaling down. The heat flux concomitantly rises up due to the shrinking dimension, and severe hot spots are formed [1]. The problem of thermally induced failure gradually becomes a bottleneck restricting the further development of microelectronic technology. Therefore, thermal management is particularly important today [2,3]. 

Compared with the conventional thermal management technologies, the micro-channel based heat sink has outstanding heat dissipation performance [4,5,6]. Fabricated using micro processing, the micro-channel heat sink can be integrated with semiconductor chips, and it is becoming one of the most promising chip-level cooling methods today [7,8]. Previous research shows that the micro-channel heat sink performance is influenced by various factors [9]. The shape of the micro-channels and their configuration may largely affect the cooling performance [10,11]. In terms of the micro-channel aspect ratio and inner surface roughness, higher aspect ratio and larger surface roughness are both preferable for heat dissipation [12,13]. In order to study the effects of disturbance in the micro-channels, researchers build turbulent micro structures in the micro-channel, which improve the heat exchange efficiency and realize the consistency of flow rate [14,15]. The liquid working as coolant for micro-channel is also studied, and the heat capacity, thermal conductivity and viscosity all significantly affect the cooling capacity of the micro-channel [16,17]. In addition, the phase change mechanism may be employed to realize a two-phase micro fluid channel heat sink, which further improves its heat dissipation capability [18]. 

Unfortunately, most of these studies do not look into the detail of micro-channel heat sink, and the understanding of such a device is quite limited [19]. This is mainly because few tools or methods are available for directly monitoring details inside the micro-channel [20]. The discrete temperature sensors can only be placed at the inlet and outlet of the heat sink, while the infrared (IR) camera can only take the temperature distribution images of the heat sink surface [21,22]. Neither of these methods can provide the details inside the micro-channels. In addition, some researches are based on simulation, where the conclusion can only be some non-proven hypothesis [23,24]. 

In order to overcome these limitations, a microfluidic channel based heat sink with integrated temperature sensors is proposed in this paper. The thin-film temperature sensors are fabricated together with the micro-channel heat sink, which realizes the high-spatial-resolution monitoring of the temperature inside the micro-channels. Benefiting from the high sensitivity and accuracy of these integrated sensors, the temperature profile along the micro-channel is extracted, and even small temperature perturbations can be observed. The micro-channel heat sink behaviors under different conditions are studied in depth using such sensors. Experimental results not only prove the outstanding heat dissipation performance of the micro-channel heat sink, but also match with simulation results quite well, revealing a series of phenomenon not observed before. This could be a powerful tool for study of a micro-channel based heat sink.

## 2. Design Considerations 

**Figure 1:**
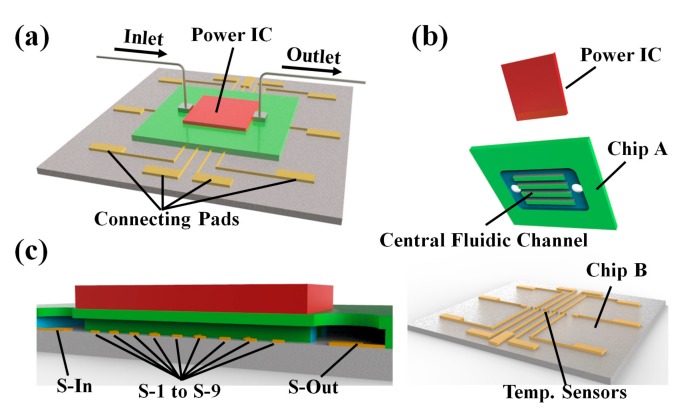
(**a**) The 3-D schematic illustration of the micro-channel based heat sink with integrated thin film temperature sensors and simulated heat source; (**b**) The structure of the heat sink 3D breakdown drawing; (**c**) Sectional view along the inlet to outlet in the direction of heat sink, where the location of thin film temperature sensors (S-In to S-Out).

The proposed microfluidic channel based heat sink with integrated temperature sensors is shown in Figure 1a. The heat sink is mainly composed of three parts, including the power IC, Chip A and Chip B, shown in Figure 1b. 

A platinum (Pt) thin film coil serves as the heat source, acting as the simulated power IC. Chip A is the microfluidic channel chip. It consists of 50 straight fluidic channels, and the dimension of each channel is 50 μm × 300 μm × 5000 μm (the width is 50 μm, the depth is 300 μm and the length is 5000 μm). The channel width is determined as 50 μm as the tradeoff between fluidic resistance and size reduction. In order to achieve a considerable heat dissipation performance, the microfluidic channels cover the entire power IC (4000 μm × 4000 μm). The coolant flows through the micro-channels and removes the heat from the power IC. The thin film temperature sensors are fabricated on the surface of Chip B and correspond with the central fluidic channel. The cross-sectional picture (Figure 1c) illustrates that two temperature sensors, named S-In and S-Out, are placed at the inlet and outlet of the heat sink, respectively. Nine temperature sensors, denoted as S1 to S9, are equally distributed in the central fluidic channel, with the pitch of 500 μm. This configuration ensures that the temperature profile in the fluidic channel can be extracted. A Pt thin film based resistor is employed as the temperature sensor, because of its high sensitivity and stability. The temperature coefficient of resistance (TCR) of the annealed Pt thin film remains stable even at elevated temperatures. To precisely measure the resistance of the temperature sensor, all sensors are 4-wire connected, eliminating the effect of parasitic resistance of the connection wires.

## 3. Fabrication and Measurement Setup 

**Figure 2:**
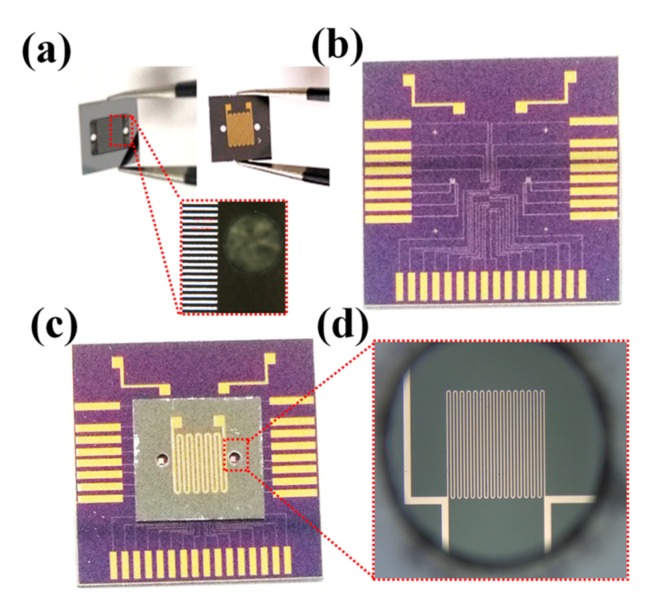
(**a**) shows the microfluidic channel Chip (Chip A); (**b**) Chip B integrated with temperature sensors and; (**c**) is the micro-channel heat sink after bonding; and (**d**) is the microscopic picture of the temperature sensor through the outlet.

Chip A is firstly fabricated using the etching process. The inlet and outlet are drilled using an ultraviolet (UV) laser. After that, titanium (Ti) (20 nm)/Pt (200 nm) thin film are deposited and patterned on the front surface of Chip A to form the heat source, where the Ti thin film serves as an adhesion layer. The thin film resistor temperature sensors are fabricated on Chip B. A thin silicon nitride (SiN) layer (1 μm) is grown on the silicon substrate for insulation purposes. The Ti (20 nm)/Pt (200 nm) thin film are deposited and patterned to form the resistor temperature sensors. The Chip B is then annealed at 350 °C for 1 h. The Chip A and Chip B are aligned and die-to-die bonded using 2 μm benzocyclobutene (BCB) epoxy. The BCB epoxy as the bonding layer also works as a protective layer. The BCB epoxy after curing is high-strength and resistant to abrasion, which prevents the Pt thin film temperature sensors from contacting the coolant directly. Such protecting methods ensure the reliability of the Pt temperature sensors. Figure 2a shows the fabricated Chip A, as well as the heat source on the front side. The Chip B after annealing is shown in Figure 2b, the thin-film temperature sensors and connecting pads are deposited and patterned on it. Figure 2c shows the whole microfluidic channel-based heat sink after bonding, and the last picture (Figure 2d) is the microscope picture, which shows that the outlet is well aligned with the corresponding thin-film temperature sensor.

**Figure 3:**
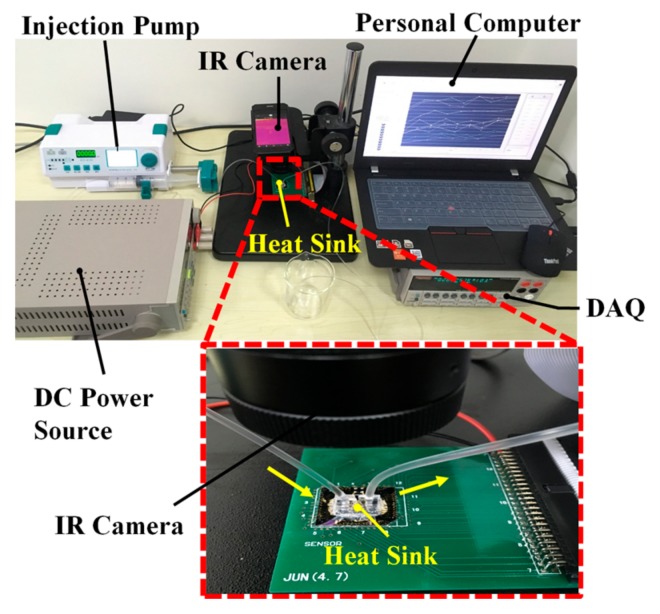
The physical picture of thermal testing system.

The as-fabricated device is mounted and wire-bonded on a customized printed circuit board (PCB). A thermal testing system is built up for real-time measurement. As shown in Figure 3, the heat source is driven by a direct-circuit (DC) power source, while the coolant (deionized water) flow rate is controlled by an injection pump. All the sensors are connected to the customized data acquisition system (DAQ), and a Labview program is developed for automatic measurement and data process. The resistance changes of the sensors are acquired one by one with a time step of 50 ms, and are then transmitted to PC for temperature conversion, display and storage. An infrared (IR) camera monitors the surface temperature of power IC in real time. 

## 4. Results and Discussion

**Figure 4:**
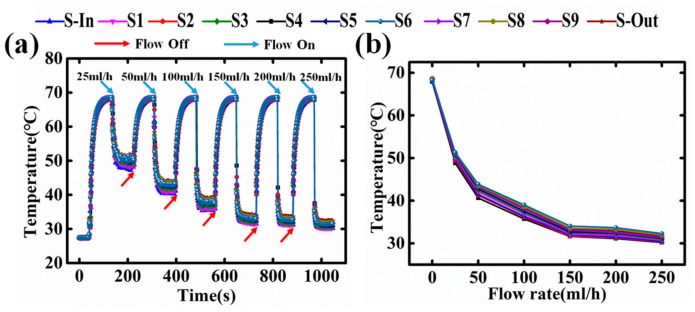
(**a**) Temperature sensors response under different flow rate (25 mL/h, 50 mL/h, 100 mL/h, 150 mL/h, 200 mL/h, 250 mL/h) with the condition of flow off or flow on; (**b**) The temperature profile in the micro-fluid channel under different flow rates.

The heat source resistance after annealing is measured as 90 Ω, which is a reasonable value for a DC power source to drive. Figure 4a shows the transient responses of all sensors, with different flow rates (25 mL/h, 50 mL/h, 100 mL/h, 150 mL/h, 200 mL/h, 250 mL/h) but a constant heating power of 1.5 W. Each temperature sensor accurately reads a stable micro fluid temperature as 69.2 °C without coolant flow. When the coolant flow is turned on, the temperature drops sharply, showing a very fast cooling speed. In contrast, when the coolant flow is turned off, it takes about 10 s for the temperature to recover. This could possibly be attributed to the relatively high heat capacity of water, where the power IC needs more time to heat up the micro-channels full of coolant. Such a result implies that even if the coolant flow is stopped due to unexpected reasons, there is enough time to cut off the power before the power IC reaches its failure temperature. Figure 4b plots the temperature relation with flow rate. The temperature does not decrease linearly with flow rate, but eventually saturates at 31 °C. This is very close to the room temperature (≈28 °C), revealing that the heat resistance for the micro-channel heat sink is considerably small.

**Figure 5:**
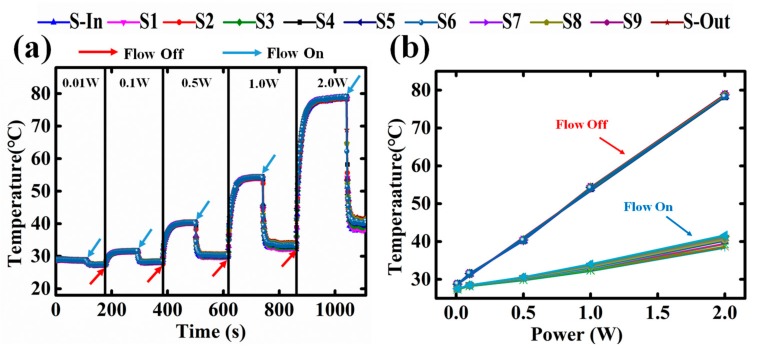
(**a**) Temperature sensors response under different power applied on the heat source (0.01 W, 0.1 W, 0.5 W, 1.0 W, 2.0 W) with the condition of flow off or flow on; (**b**) The temperature profile in the micro-fluid channel from the inlet to outlet under different heating power.

Figure 5a shows the coolant flow (100 mL/h) on/off transient responses under different heating powers (0.01 W, 0.1 W, 0.5 W, 1.0 W, 2.0 W). The temperature recovery time does not show significant difference as heating power increases. Figure 5b shows the temperature curves with heating power. Both curves increase linearly; however, the flow-on curve slope is much lower than that of the flow-off curve. The temperature difference of 2 W input power is more than 40 °C. Considering the low coolant flow rate (100 mL/h, or 1.67 mL/min), the heat dissipation performance of the micro-channel heat sink is quite considerable.

**Figure 6:**
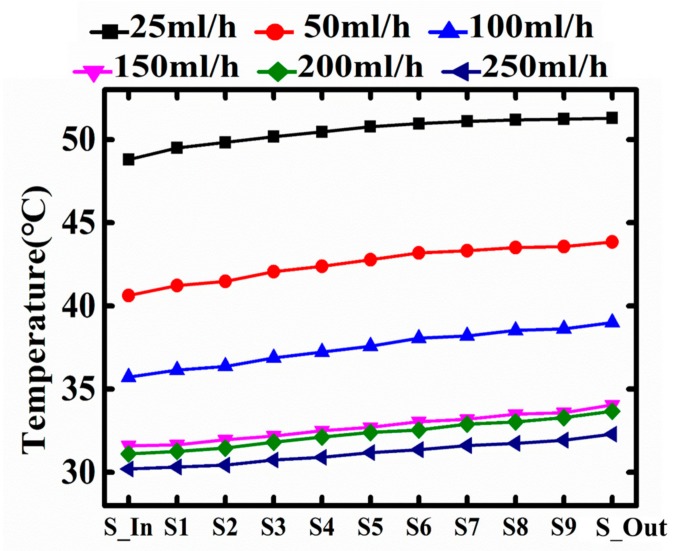
Under different flow rate, the temperature profile in the micro-fluid channel from the inlet to outlet where corresponding the temperature sensors from S-In to S-Out.

**Figure 7:**
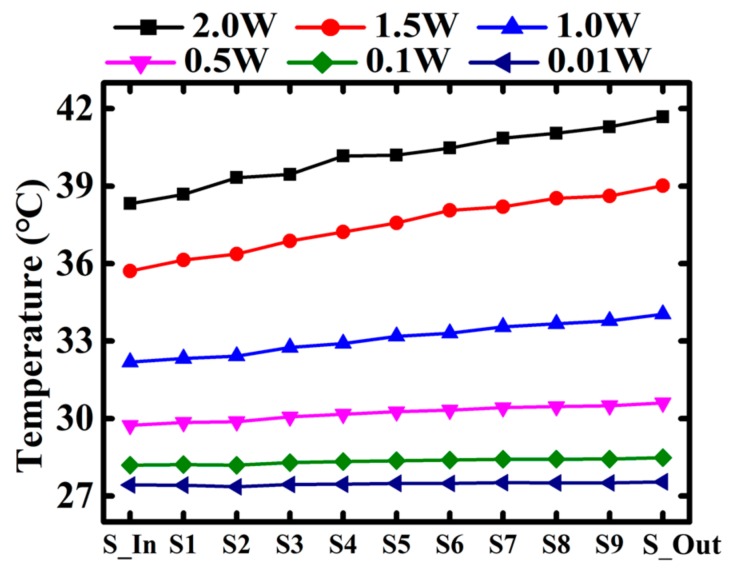
Under different heating power, the temperature profile in the micro-fluid channel from the inlet to outlet where corresponding the temperature sensors from S-In to S-Out.

It is worth noting that the flow-on curves split in Figure 6 and Figure 7, meaning the local temperatures along the micro-channel are not uniform. Figure 6 shows the temperature profiles along the central fluidic channel with different flow rates (input power at 1.5 W). In general, the temperature increases from the inlet to outlet. The experimental results also verify the simulation results of Qu et al. [24]. The slope of temperature profile curves remains constant despite of the change of flow rate, while the curve parallel shifts downwards as the flow rate increases. 

The temperature profiles along the micro-channel with different input power are plotted and shown in Figure 7. When the input power is small, the temperature at the inlet to outlet are almost the same. While the input power keeps increasing, the temperature profile presents a growing trend from inlet to outlet. The temperature difference between inlet and outlet is lifted to 3 °C for 2 W input power. 

Comparing the curves under the two different conditions, it is found that the slope of the temperature profile is solely related to the input power, and independent of the coolant flow rate. For practical use of the micro-channel heat sink, the temperature non-uniformity could be a severe problem if the heating power is large enough. Such temperature non-uniformity may have an undesired influence on power IC, or even make the power IC malfunction. Controlling the maximum temperature of power IC thus may not be sufficient, and its temperature non-uniformity must be taken into account. Moreover, since the slope of the temperature profile is independent of the coolant flow rate, simply increasing the flow rate can only suppress the maximum temperature, but not relieve the temperature non-uniformity issue. At this point, the original understanding is not sufficient. Minimizing such temperature non-uniformity may rely on the innovative design of the micro-channel based heat sink.

**Figure 8:**
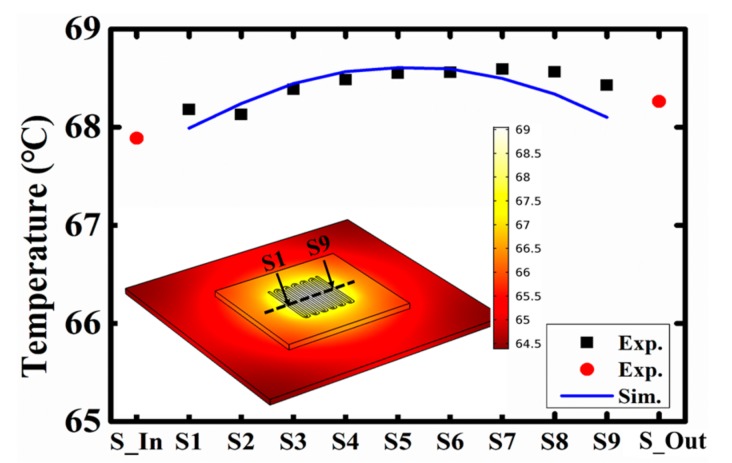
Measured and simulated spatial temperature distribution of the heat sink under the condition of flow is off.

**Figure 9:**
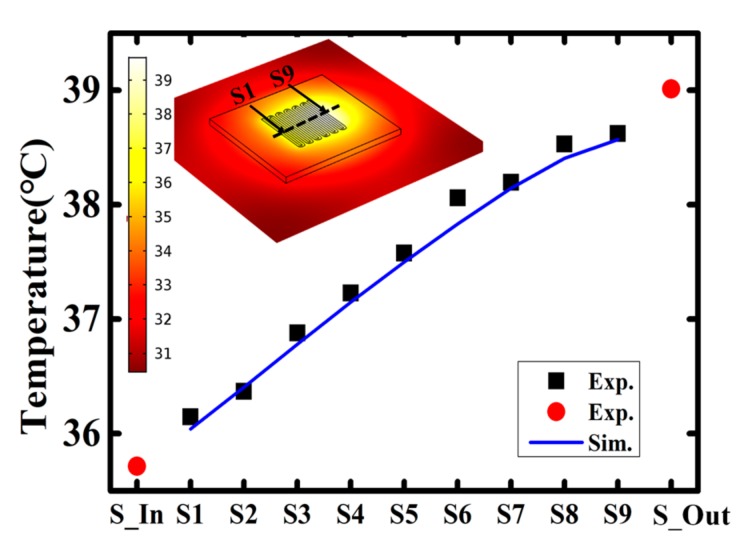
Measured and simulated spatial temperature distribution of the heat sink under the condition of flow is 100 mL/h.

Figure 8 and Figure 9 show the finite elements modeling (FEM) simulation results of the micro-channel heat sink. When the coolant flow is off, the temperature profile obeys a Gaussian distribution, where the temperature peak is located at the center of the microchannel heat sink. The experimental results agree well with the simulated curve. In particular, with the coolant flow rate increases, the peak of temperature shifts from the center to the outlet (the flow direction) of the microchannel heat sink. When the coolant flow rate is fixed at 100 mL/h, as shown in Figure 9, the peak temperature shifts to the area near the outlet. This is also proven by the experimental results. Therefore, the integrated thin film temperature sensors can be used to directly verify the modeling results, and help to build a convincing simulation model.

**Figure 10:**
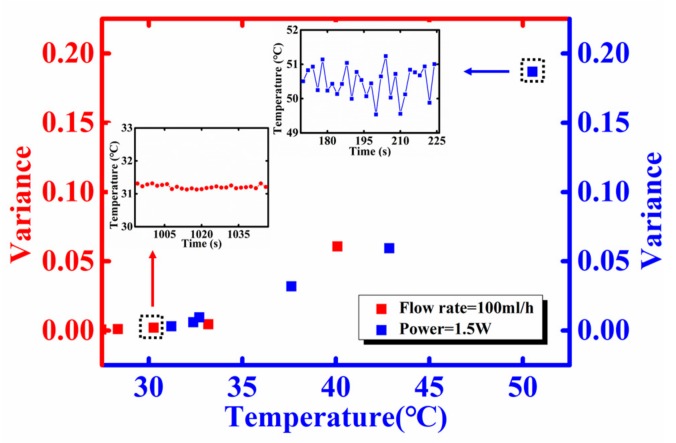
The variance of temperature fluctuation with the temperature change of the micro fluid by changing the power on power IC or the flow rate.

A noteworthy phenomenon is illustrated in Figure 10. The variance of temperature fluctuation along the micro-channel is more obvious as the temperature increases. Regardless of changing the input power or the flow rate, as long as the temperatures are approximately equal, the corresponding fluctuations in the micro-channel are similar. The curve shows that the temperature fluctuation is within the range of 0.2 °C when the micro fluid temperature is relatively low (31.2 °C). However, when the temperature increases to a higher value (≈50.5 °C), its fluctuation range becomes greater than 1.5 °C. The result implies that temperature fluctuation in the microchannel heat sink is related to temperature instead of flow rate or input power. The reason for such a phenomenon could possibly be the gas separated out from the coolant. The air solubility in water decreases when the temperature rises, and thus amount of micro gas bubbles may form in the heated micro-channels, which increase the fluctuation of temperature. Further study of the temperature rated fluctuation will constitute future work.

## 5. Conclusions 

In this paper, a microfluidic channel based heat sink with integrated temperature sensors has been simulatively and experimentally investigated. The conclusions obtained from this study are summarized as follows:

(1) The integrated thin film temperature sensors can monitor the temperature in the channels accurately in real time, and the resolution is better than 0.1 °C. Due to their small size, the temperature profile within the micro-channel can be obtained with high resolution, high stability and high sensitivity.
(2) The experimental results match well with simulation results and can be used to directly verify the modeling results, helping to build a convincing simulation model.
(3) The slope of the temperature distribution trend in the micro-channel from inlet to outlet is related to the heating power and independent of the flow rate. Such temperature non-uniformity may bring problems for power IC and the micro-channel based heat sink should be designed and optimized for minimizing such temperature non-uniformity.
(4) As the temperature increases, the temperature fluctuation is more obvious. The cause of the phenomenon is possibly the phase change or the decreasing of air solubility in water when the temperature rises. The integrated sensors could be a powerful tool for studying the micro-channel based heat sink.
